# *CSF3R* T618I mutant chronic myelomonocytic leukemia (CMML) defines a proliferative CMML subtype enriched in *ASXL1* mutations with adverse outcomes

**DOI:** 10.1038/s41408-021-00449-9

**Published:** 2021-03-11

**Authors:** Evandro D. Bezerra, Terra L. Lasho, Christy M. Finke, Antoine N. Saliba, Michelle A. Elliott, Animesh D. Pardanani, Naseema Gangat, Abhishek A. Mangaonkar, Rhett P. Ketterling, Ayalew Tefferi, Eric Solary, Mrinal M. Patnaik

**Affiliations:** 1 Division of Hematology, Mayo Clinic, Rochester, MN USA; 2 Department of Laboratory Medicine and Pathology, Mayo Clinic, Rochester, MN USA; 3 Faculte de Medecine, Universite Paris-Saclay, 94270 Le Kremlin-Bicetre, France; 4 Department of Hematology and INSERM U1287, Gustave Roussy Cancer Center, 94805 Villejuif, France

**Keywords:** Myeloproliferative disease, Myelodysplastic syndrome

Dear Editor,

Chronic myelomonocytic leukemia (CMML) is a clonal myeloid disorder characterized by peripheral blood (PB) monocytosis (absolute monocyte count (AMC) ≥1 × 10^9^/L and ≥10% of the total white blood cell count (WBC)) and overlapping features of myelodysplastic syndromes and myeloproliferative neoplasms (MPN)^1^. Sustained PB monocytosis is a hallmark of CMML, with a mutational signature consisting of biallelic *TET2*, or *TET2* and *SRSF2* co-mutations, biasing hematopoiesis toward monocytosis^2^. Chronic neutrophilic leukemia (CNL) is a rare MPN characterized by sustained mature neutrophilia (WBC ≥ 25 × 10^9^/L, with segmented neutrophils and band forms ≥ 80% of the WBC), with an AMC < 1 × 10^9^/L^3^. CNL commonly occurs due to oncogenic driver mutations involving the colony-stimulating factor receptor, *CSF3R* (90–100%)^4,5^. Among *CSF3R* mutations described in CNL, the T6181 membrane proximal point mutation activating Janus-activated kinase (JAK)/signal transducer and activator of transcription factor signaling is the most common (74%)^4,6^. These mutations potentially render CNL responsive to therapy with JAK inhibitors, such as ruxolitinib^7^. On the other hand, *CSF3R* mutations, especially *CSF3R* T618I, are very infrequent in CMML (<5%)^8,9^. We carried out this study to assess the prevalence, phenotypic features, and outcomes of patients with CMML who harbor the *CSF3R* T618I mutation.

Eight hundred and forty-six patients with CMML, defined per the World Health Organization (WHO) 2016 criteria, from Mayo Clinic, Minnesota and the Groupe Francais des Myelodysplasies were included in this study (August 1994 through July 2019). All patients underwent targeted next-generation sequencing for myeloid-relevant genes obtained at diagnosis or at first referral using previously described methods^2,10^. Categorical variables were compared by Fisher’s exact or Pearson’s chi square. Continuous variables were compared by Wilcoxon test. Overall survival (OS) was defined as the time from diagnosis to death. Acute myeloid leukemia-free survival (AML-FS) was defined as the time from diagnosis to transformation to AML or death. Patients who underwent hematopoietic cell transplantation prior to AML transformation were censored for AML-FS estimates. Both OS and AML-FS were estimated by the Kaplan–Meier method and compared by log-rank test.

We identified 6 (1%) patients who met the 2016 WHO criteria for CMML and who harbored *CSF3R* mutations, all of whom had the *CSF3R* T618I missense alteration; 3 (50%) were males, median age 63 years, with a median AMC of 6 × 10^9^/L (range 2–23). Five (100%) of five evaluable patients had bone marrow (BM) dysplasia, a finding not seen in CNL. As expected, all six *CSF3R* T618I mutant patients had a proliferative CMML phenotype, with marked neutrophilia in addition to the monocytosis. Four (100%) of the 4 evaluable patients also had circulating immature myeloid cells (myelocytes and metamyelocytes). The *CSF3R* T618I variant allele frequency (VAF) burdens were >40% in 3 (60%) of 5 evaluable cases (range 10–63%). While the *CSF3R* mutational VAF in three patients was in the heterozygous range, all three had adult-onset leukocytosis/monocytosis, making the likelihood that these variants were germline, quite low^11^. We, however, acknowledge that we did not have germline tissue for further confirmation. Three patients had a normal karyotype, while the remaining three had X chromosome abnormalities (Table 1).

**Table 1:** Individual patient’s characteristics and outcomes.

Patient	Age, years	Gender	CMML subtype		CBC						BM		Karyotype	*CSF3R* VAF %	Co-mutations (VAF %)	Treatment	Outcomes
			FAB	WHO	Hb, g/dL	Plts, ×10^9^/L	WBC, ×10^9^/L	ANC, ×10^9^/L (%)	AMC, ×10^9^/L (%)	PB blasts (%)	Blasts (%)	Dysplasia					
1	62	F	P	2	13	50	40	21 (53)	6 (15)	12	16	+	46, X, i(Xq13) [14]/46, XX [6]	10	*SRSF2* (49)	HU/induction	AML transformation in 1 month and death 9 months later
2	39	F	P	2	6	50	134	114 (85)	19 (14)	6	15	+	46, X, i(Xq13) [7]/46, XX [4]	63	*ASXL1* (35)/*STEBP1* (51)	HU	No follow-up
3	48	M	P	0	7	168	180	151 (84)	23 (13)	0	1	+	46, XY [20]	58	*ASXL1* (25)	HMA	Refractory disease and died in 20 months
4	66	M	P	0	8	60	31	25 (81)	4 (13)	0	1	+	46, XY [20]	51	*ASXL1* (45)	HU	Alive at 6 months
5	69	F	P	1	8	173	35	23 (66)	5 (14)	2	0	+	46, X, del(X) [12]/46, XX [8]	12	*ASXL1* (46)	HU	No follow-up
6	64	M	P	0	11	302	21	18 (86)	2 (11)	0	3	UK	46, XY [UK]	UK	*ASXL1* (49)/*UA2F1* (44)	HU	Died in 17 months

Individual characteristics and outcomes of CMML patients with *CSF3R* T618I mutation. Characteristics are based on diagnosis or first referral.

*M* male, *F* female, *WHO* World Health Organization, *FAB* French–American–British, *CBC* complete blood count, *Hb* hemoglobin, *WBC* white blood count, *ANC* absolute neutrophils count, *AMC* absolute monocyte count, *Plt* platelets, *PB* peripheral blood, *BM* bone marrow, *VAF* variant allele frequency, *HU* hydroxyurea, *induction* cytarabine-based intense chemotherapy regimens, *RBC* red blood cells, *AML* acute myeloid leukemia, *UK* unknown.

In comparison to *CSF3R* wild-type (WT) CMML patients, those with the *CSF3R* T618I mutation were younger (median 63 vs. 72 years; *p* = 0.0055), had lower hemoglobin values (Hb, median 8 vs. 11 g/dl; *p* = 0.0186), higher WBC (median 38 vs. 13 × 10^9^/L; *p* = 0.0013), higher percentage of neutrophils (median 83 vs. 52%; *p* = 0.001), higher AMC (median 6 vs. 2 × 10^9^/L; *p* = 0.024), lower percentage of monocytes (median 13 vs. 23%; *p* = 0.0069), higher percentage of PB blasts (median 1 vs. 0%; *p* = 0.0472), and were more likely to be classified as proliferative CMML (100 vs. 48%; *p* = 0.012). There were no *TET2* mutations in the *CSF3R* T618I mutant CMML group, vs. 59% in the WT group (*p* = 0.005), while *ASXL1* mutations were more frequent in the *CSF3R* T618I mutant group (83 vs. 41%; *p* = 0.045) in comparison to the WT group (Supplementary Table 1). While not statistically significant, 5 of the 6 (83%) *CSF3R* T618I mutant CMML patients did not have concomitant *SRSF2* mutations, with 4 of the 6 (66%) not having any splicing mutation at all.

At last follow-up, 3 (50%) and 400 (48%) deaths and 1 (17%) and 122 (15%) leukemic transformations were documented in the *CSF3R* T618I mutant and WT CMML groups, respectively. The median OS was significantly shorter in CMML patients with the *CSF3R* T618I mutation (median 1.4 vs. 3.0 years; *p* = 0.049), in comparison to the WT patients, with no difference in the AML-FS (1.4 vs. 3.2 years; *p* = 0.17) (Fig. 1). None of our patients received ruxolitinib or underwent allogeneic stem cell transplantation. Five of the six patients were treated with hydroxyurea and one patient was treated with hypomethylating agent therapy, with no response.

**Fig. 1:**
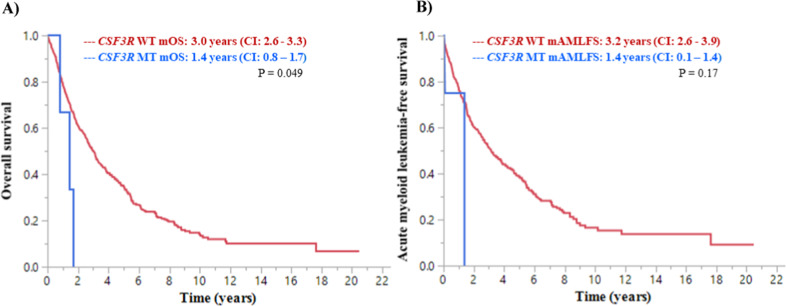
Kaplan–Meier survival analysis of CMML patients stratified by the presence or absence of the *CSF3R*T618I mutation. **A** Inferior overall survival of *CSF3R*T618I mutant CMML in comparison to *CSF3R* wildtype patients. **B** Inferior acute myeloid leukemia-free survival of *CSF3R*T618I mutant CMML in comparison to *CSF3R* wildtype patients.

Given the higher prevalence of *ASXL1* mutations in the *CSF3R* T618I mutated cohort, and the known detrimental prognostic impact of *ASXL1* mutations in CMML, we performed a subgroup analysis comparing *CSF3R* T618I mutant CMML with *ASXL1* mutant CMML patients (Supplementary Table 1)^10^. In this comparison, once again, *CSF3R* mutant CMML patients were more likely to be younger in age (*p* = 0.009), have lower Hb levels (*p* = 0.005), have higher WBC (*p* = 0.009), have lower AMC (*p* = 0.012), and were less likely to have *TET2* mutations (*p* = 0.029). There was no OS difference between the two groups (Supplementary Fig. 1).

In summary, we present a cohort of patients who met 2016 WHO criteria for CMML and who harbored an oncogenic driver mutation in *CSF3R* T618I. While this mutation is fairly specific to CNL, infrequent occurrences in other myeloid neoplasms have been documented^8,9^. In our opinion, in the context of accurate histopathological analyses, the true frequency of these mutations in CMML is <1%. In addition, the *CSF3R* T618I mutation when present defines a unique proliferative CMML subtype characterized by pronounced leukocytosis/neutrophilia/monocytosis, with a unique molecular signature (*ASXL1*mt/*TET2*wt), with infrequent splicing mutations. The high frequency of X chromosome abnormalities remains to be defined. We have in the past demonstrated the negative prognostic impact of the *ASXL1*mt/*TET2*wt genotype in CMML, a finding once again validated by the poor OS of this group in the current study (median OS 1.4 years)^2,12^. In fact, the molecular landscape of *CSF3R* T618I mutant CMML is more akin to *BCR-ABL1*-negative atypical chronic myeloid leukemia (CML), where *ASXL1* mutations are frequent (80%) and splicing mutations are less common (30%)^13^. However, atypical CML is not associated with monocytosis, and given the fact that in CMML biallelic *TET2* or *TET2/SRSF2* co-mutations skew hematopoiesis toward monocytosis, the etiology of monocytosis in these patients remains to be elucidated. While the presence of BM dysplasia along with concomitant monocytosis/neutrophilia morphologically distinguishes *CSF3R* T618I mutant CMML from CNL, further work is needed to see whether *CSF3R* T618I mutant CMML is truly a CMML subtype, or if akin to *PDGFRA/B* and *FGFR1* rearranged myeloid neoplasms, it deserves its own classification schema as a *CSF3R* T618I mutant myeloid neoplasm with monocytosis.

## Supplementary information

Supplementary table 1

Supplementary figure 1
