# Supplementary material for: CSF3R T618I mutant chronic myelomonocytic leukemia (CMML) defines a proliferative CMML subtype enriched in ASXL1 mutations with adverse outcomes
Source: Blood Cancer J. 2021 Mar 11;11(3):54. doi: 10.1038/s41408-021-00449-9 (PMC7952717; doi:10.1038/s41408-021-00449-9)
Supplement: Supplementary file 1 — Supplementary table 1 [file 41408_2021_449_MOESM1_ESM.docx]

**Individual characteristics of CMML patients with CSF3R T618I mutation**

**Supplementary Table 1. Patient’s characteristics and outcomes.**

| **Variables [Median or n; range or %]** | **CMML without *CSF3R* mutations** | **CMML with *CSF3R* (T618I) mutations** | **P value***** | **ASXL1 mutated CMML without *CSF3R*** | **ASXL1 mutated CMML with *CSF3R* (T618I)** | **P value***** |
| --- | --- | --- | --- | --- | --- | --- |
| **N (%)** | 840 (99) | 6 (1) |  | 341 (41) | 5 (1) |  |
| **Male, N (%)** | 547 (68) | 3 (50) | 0.39 | 231 (73) | 3 (60) | 0.62 |
| **Age years; median (range)** | 72 (18-93) | 63 (39-69) | **0.0055** | 73 (27-93) | 64 (39-69) | **0.0093** |
| **Hb g/dL; median (range)** | 11 (5-18) | 8 (6-13) | **0.0186** | 11 (5-18) | 8 (6-11) | **0.0052** |
| **WBC x 10^9^/L; median (range)** | 13 (2-367) | 38 (21-180) | **0.0013** | 15 (2-367) | 35 (21-180) | **0.0098** |
| **ANC %; median (range)** | 52 (2-85) | 83 (53-86) | **0.001** | N/A | N/A | N/A |
| **AMC x 10^9^/L; median (range)** | 2 (1-84) | 6 (2-23) | **0.024** | 3 (1-84) | 5 (2-23) | 0.13 |
| **AMC %; median (range)** | 23 (10-86) | 13 (11-15) | **0.0069** | 22 (10-86) | 13 (10-14) | **0.0125** |
| **Plts x 10^9^/L; median (range)** | 107 (3-1,264) | 114 (48-302) | 0.93 | 101 (9-1,264) | 168 (48-302) | 0.67 |
| **PB IMC N (%)** | 400 (56) | 4 (100) | 0.14 | 179 (60) | 3 (100) | 0.28 |
| **PB blasts %; median (range)** | 0 (0-19) | 1 (0-12) | **0.0472** | 0 (0-19) | 0 (0-6) | 0.42 |
| **BM blasts %; median (range)** | 4 (0-19) | 2 (0-16) | 0.71 | 4 (0-19) | 1 (0-15) | 0.29 |
| **BM dysplasia N (%)** | UK | 5 (83) | N/A | UK | 4 (80) | N/A |
| **WHO 2016 diagnosis**  **CMML-0**  **CMML-1**  **CMML-2** | 456 (54)  228 (27)  156 (19) | 3 (50)  1 (17)  2 (33) | 0.62 | 188 (55)  84 (25)  68 (20) | 3 (60)  1 (20)  1 (20) | 0.064 |
| **FAB CMML**  **proliferative diagnosis** | 399 (48) | 6 (100) | **0.0126** | 200 (59) | 5 (100) | 0.07 |
| **Abnormal karyotype** | 201 (28) | 3 (50) | 0.36 | 81 (27) | 2 (40) | 0.62 |
| **Mayo-French Karyotype risk**  **Low;**  **Intermediate;**  **High;** | 564 (78)  135 (19)  25 (4) | 3 (50)  3 (50)  0 | 0.14 | 230 (77)  58 (20)  9 (3) | 3 (60)  2 (40)  0 | 0.50 |
| **NGS data; n (%)**   1. **Epigenetic regulators**   ***TET2***  ***IDH1***  ***IDH2***  ***DNMT3A***   1. **Chromatin regulators**   ***ASXL1***  ***EZH2***   1. **Spliceosome factors**   ***SRSF2***  ***SF3B1***  ***U2AF1***  ***ZRSR2***   1. **Cell signaling**   ***NRAS***  ***KRAS***  ***CBL***  ***JAK2***  ***KIT***  ***FLT3***  ***NPM1***   1. **Tumor suppressor gene**   ***TP53***   1. **Others**   ***SETBP1*** | 492 (59)  9 (1)  44 (5)  41 (5)  341 (41)  41 (5)  369 (44)  38 (5)  54 (6)  41 (5)  128 (15)  83 (10) 114 (14)  63 (8)  22 (3)  19 (2)  10 (2)  20 (3)  53 (6) | 0  0  0  0  5 (83)  0  1 (17)  0  1(17)  0  0  0  0  0  0  0  0  0  1 (17) | **0.0049**  1.0  1.0  1.0  **0.0456**  1.0  0.24  1.0  0.39  1.0  0.60  1.0  1.0  1.0  1.0  1.0  1.0  1.0  0.33 | 172 (51)  4 (1)  23 (7)  10 (3)  341 (100)  29 (9)  167 (49)  6 (2)  31 (9)  12 (4)  66 (19)  29 (9)  61 (18)  23 (7)  13 (4)  6 (2)  1 (0.4)  4 (1)  32 (9) | 0  0  0  0  5 (100)  0  0  0  1 (20)  0  0  0  0  0  0  0  0  0  1 (20) | **0.0299**  1.0  1.0  1.0  N/A  1.0  0.0612  1.0  0.39  1.0  0.59  1.0  0.59  1.0  1.0  1.0  1.0  1.0  0.40 |
| ***CSF3R* VAF; median (range)** | N/A | 51 (10-63) | N/A | N/A | 55 (10-63) | N/A |
| **Mayo Molecular Model risk**  **Low;**  **Intermediate-1;**  **Intermediate-2;**  **High;** | 79 (11)  210 (29)  226 (32)  200 (28) | 0  0  1 (25)  3 (75) | 0.19 | 0  45 (15)  105 (35)  148 (50) | 0  0  0  3 (100) | 0.22 |
| **HMA, N (%)** | 245 (29) | 1 (17) | 0.68 | 120 (35) | 1 (20) | 0.66 |
| **Hydroxiureia, N (%)** | 182 (22) | 5 (83) | **0.0025** | 95 (28) | 4 (80) | **0.0249** |
| **Induction, N (%)** | 52 (6) | 1 (17) | 0.32 | 23 (7) | 0 | 1.0 |
| **RBCs transfusions, N (%)** | 238 (28) | 4 (67) | 0.0593 | 112 (33) | 3 (60) | 0.34 |
| **Plts transfusions, N (%)** | 178 (21) | 1 (17) | 1.0 | 81 (24) | 0 | 0.60 |
| **HCT, N (%)** | 57 (7) | 0 | 1.0 | 26 (8) | 0 | 1.0 |
| **AML N (%)** | 122 (15) | 1 (17) | 1.0 | 54 (16) | 0 | 1.0 |
| **Death N (%)** | 400 (48) | 3 (50) | 1.0 | 183 (54) | 2 (40) | 0.67 |
| **F/U months, median (range)** | 12 (0-246) | 7 (0.4-17) | 0.26 | 12 (0-212) | 6 (0.4-17) | 0.31 |

**Supplementary table 1.**  Characteristics and outcomes of CMML patients with or without *CSF3R* T618I mutation. Characteristic are based on diagnosis or 1^st^ referral. ^*^P value calculated by chi-square for categorical variables and Wilcoxon test for continuous variables. Hb = hemoglobin, WBC = white blood count, ANC = absolute neutrophils count, AMC = absolute monocyte count, Plt = platelets, PB = peripheral blood, IMC = immature myeloid cells, BM = bone marrow, WHO = world health organization, FAB = French American British, NGS = next generation sequencing, VAF = variant allele frequency, HMA = hypomethylant agents, induction = Cytarabine based intense chemotherapy regimens, RBCs = red blood cells, HCT = hematopoietic cell transplant, AML = acute myeloid leukemia transformation, F/U = follow-up.
