# Supplementary material for: CSF3R T618I mutant chronic myelomonocytic leukemia (CMML) defines a proliferative CMML subtype enriched in ASXL1 mutations with adverse outcomes
Source: Blood Cancer J. 2021 Mar 11;11(3):54. doi: 10.1038/s41408-021-00449-9 (PMC7952717; doi:10.1038/s41408-021-00449-9)
Supplement: Supplementary file 2 — Supplementary figure 1 [file 41408_2021_449_MOESM2_ESM.docx]

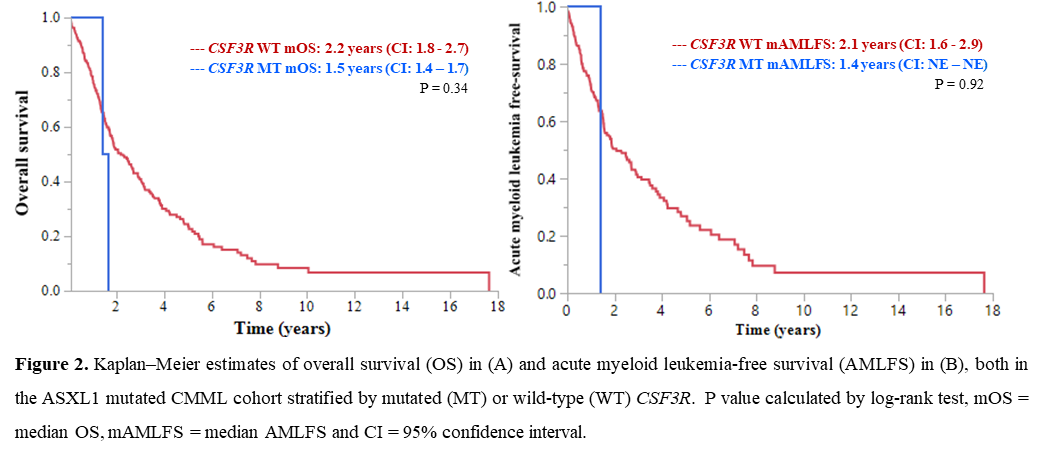


**A)**

**B)**

**Individual characteristics of CMML patients with CSF3R T618I mutation**

**Supplementary figure 1.** Kaplan–Meier estimates of overall survival (OS) in (A) and acute myeloid leukemia-free survival (AMLFS) in (B), both in the ASXL1 mutated CMML cohort stratified by mutated (MT) or wild-type (WT) *CSF3R*. P value calculated by log-rank test, mOS = median OS, mAMLFS = median AMLFS and CI = 95% confidence interval.
